# β_2_-Adrenergic Receptor-Dependent Attenuation of Hypoxic Pulmonary Vasoconstriction Prevents Progression of Pulmonary Arterial Hypertension in Intermittent Hypoxic Rats

**DOI:** 10.1371/journal.pone.0110693

**Published:** 2014-10-28

**Authors:** Hisashi Nagai, Ichiro Kuwahira, Daryl O. Schwenke, Hirotsugu Tsuchimochi, Akina Nara, Tadakatsu Inagaki, Sayoko Ogura, Yutaka Fujii, Keiji Umetani, Tatsuo Shimosawa, Ken-ichi Yoshida, James T. Pearson, Koichi Uemura, Mikiyasu Shirai

**Affiliations:** 1 Department of Forensic Medicine, Tokyo Medical and Dental University, Tokyo, Japan; 2 Department of Forensic Medicine, The University of Tokyo, Tokyo, Japan; 3 Department of Pulmonary Medicine, Tokai University Tokyo Hospital, Tokyo, Japan; 4 Department of Physiology-Heart Otago, University of Otago, Dunedin, New Zealand; 5 Department of Cardiac Physiology, National Cerebral and Cardiovascular Center Research Institute, Osaka, Japan; 6 Division of Laboratory Medicine, Department of Pathology and Microbiology, Faculty of Medicine, Nihon University School of Medicine, Tokyo, Japan; 7 Japan Synchrotron Radiation Research Institute, Hyogo, Japan; 8 Department of Clinical Laboratory Medicine, The University of Tokyo, Tokyo, Japan; 9 Department of Forensic Medicine, Tokyo Medical University, Tokyo, Japan; 10 Monash Biomedical Imaging Facility and Department of Physiology, Monash University, Melbourne, Clayton, Victoria, Australia; 11 Australian Synchrotron, Clayton, Victoria, Australia; University of Pittsburgh School of Medicine, United States of America

## Abstract

In sleep apnea syndrome (SAS), intermittent hypoxia (IH) induces repeated episodes of hypoxic pulmonary vasoconstriction (HPV) during sleep, which presumably contribute to pulmonary arterial hypertension (PAH). However, the prevalence of PAH was low and severity is mostly mild in SAS patients, and mild or no right ventricular hypertrophy (RVH) was reported in IH-exposed animals. The question then arises as to why PAH is not a universal finding in SAS if repeated hypoxia of sufficient duration causes cycling HPV. In the present study, rats underwent IH at a rate of 3 min cycles of 4–21% O_2_ for 8 h/d for 6w. Assessment of diameter changes in small pulmonary arteries in response to acute hypoxia and drugs were performed using synchrotron radiation microangiography on anesthetized rats. In IH-rats, neither PAH nor RVH was observed and HPV was strongly reversed. Nadolol (a hydrophilic β_1, 2_-blocker) augmented the attenuated HPV to almost the same level as that in N-rats, but atenolol (a hydrophilic β_1_-blocker) had no effect on the HPV in IH. These β-blockers had almost no effect on the HPV in N-rats. Chronic administration of nadolol during 6 weeks of IH exposure induced PAH and RVH in IH-rats, but did not in N-rats. Meanwhile, atenolol had no effect on morphometric and hemodynamic changes in N and IH-rats. Protein expression of the β_1_-adrenergic receptor (AR) was down-regulated while that of β_2_AR was preserved in pulmonary arteries of IH-rats. Phosphorylation of p85 (chief component of phosphoinositide 3-kinase (PI3K)), protein kinase B (Akt), and endothelial nitric oxide synthase (eNOS) were abrogated by chronic administration of nadolol in the lung tissue of IH-rats. We conclude that IH-derived activation of β_2_AR in the pulmonary arteries attenuates the HPV, thereby preventing progression of IH-induced PAH. This protective effect may depend on the β_2_AR-Gi mediated PI3K/Akt/eNOS signaling pathway.

## Introduction

Sleep apnea syndrome (SAS) is known as a major and independent risk factor for cardiovascular disease, such as systemic hypertension, myocardial infarction, cerebrovascular dysfunction, and idiopathic sudden death [1]. Repeated intermittent hypoxia (IH) is observed as cycling short time desaturations in SAS during the sleep period [2], which induces repeated increases in pulmonary arterial pressure, suggesting repeated hypoxic pulmonary vasoconstriction (HPV) occurs [3], [4]. IH-derived repeated HPV is thought to contribute to the pulmonary arterial hypertension (PAH) in SAS [3], [4]. Patients with SAS exhibited large swings in pulmonary arterial pressure during apneic events, and generally a progressive increase in pressure overnight with systolic pulmonary pressure often reaching 50–60 mmHg [4]. In general, the mechanisms of IH-induced PAH are considered to parallel those associated with chronic sustained hypoxia, and include pulmonary vasoconstriction, vascular remodeling and polycythemia [3]. However, HPV may play a more important role in the progression of PAH than the other mechanisms. Sylvester et al. estimated that HPV accounts for ∼60% of the PAH caused by SAS, and ∼40% must be due to other mechanisms [4]. Meanwhile, it has been reported that the prevalence of PAH is estimated to be less than 20% [1] or 21 to 43% in SAS [4], and that in most cases, PAH is mild [2], [5]. The question then arises as to why PAH is not a universal finding in SAS if repeated hypoxia of sufficient duration causes cycling HPV [1].

Because IH is thought to play a major role in the development of cardiovascular disorders in SAS [6], animals exposed to IH are widely used for SAS model. In the previous reports of IH animals, the degree of right ventricular hypertrophy (RVH), which was correlated with the level of PAH is mild [7] or even non-existant [8]–[16]. These results suggest that PAH is not a general finding in the IH-exposed animals.

In pulmonary arteries of normal rats, the β_1_- and β_2_-adrenergic receptors (AR) primarily exert a dilatatory effect [17]–[19]. We have previously reported that sympathoadrenal activation occurs in response to acute hypoxic exposure which acts to attenuate the locally-induced HPV through a βAR-mediated pulmonary vasodilator mechanism. This neural mechanism protects the right ventricle from pressure overload [20], [21]. Additionally, IH exposure to healthy humans and animals causes prolonged activation of the sympathoadrenal system [3], [22]–[26]. It has been reported that prolonged activation of β_2_AR switches G-protein from Gs to Gi [27]. β_2_AR-Gi-dependent activation of phosphoinositide 3-kinase (PI3K)/protein kinase B (Akt)/endothelial nitric oxide synthase (eNOS) signaling probably induces NO-dependent β_2_AR-mediated relaxation of the pulmonary artery [17]. These findings lead us to propose that in the IH-exposed animals, prolonged sympathoadrenal activation potentiates the βAR-Gi-mediated vasodilator mechanism to attenuate the magnitude of HPV, and thus, beneficially impedes the progression of PAH.

To test this hypothesis, we directly measured dynamic changes in the internal diameter (ID) of the small pulmonary arteries (100–500 µm) which are the target sites of HPV [28], using the synchrotron radiation (SR) microangiography [29] in IH-rats *in vivo*. To elucidate which βAR subtype contributes to modify HPV, the ID changes in response to acute hypoxia were measured with and without administration of atenolol (hydrophilic β_1_AR-selective β-blocker) and nadolol (hydrophilic non-selective β_1_ and β_2_AR β-blocker). Moreover, whether chronic administration of β-blockers during IH exposure promotes the PAH progression was investigated. In addition, the effects of IH on the expression level of βAR subtypes in pulmonary arteries and the downstream components of β_2_AR-mediated signaling pathway in the lung tissue were assessed. Our results may explain the low prevalence of PAH in SAS patients and inconsistencies in the development of PAH during IH-animals.

## Materials and Methods

### Animals

Experiments were conducted on 7 wk old male Sprague-Dawley rats. All rats were on a 12∶ 12-h light-dark cycle at 25°C and were provided with food and water *ad libitum*. Rats were divided into two groups. One was housed in normoxic conditions (N-rats). Another was continuously housed in an original customized hypoxic chamber with intermittent hypoxic exposure for 6 weeks, except for a 10 minute interval every fifth day when chambers were cleaned (IH-rats). The hypoxic gas mixture (prepared from N_2_ and air) and compressed air were alternately delivered to the hypoxic chamber for 90 seconds during daylight hours. The O_2_ concentration was decreased to 4% approximately every 90 seconds. Exposure was performed 8 hours/day (9∶00 AM −5∶00 PM) for 42 consecutive days. All experiments were approved by the Institutional Animal Care and Use Committee of the University of Tokyo.

### Anesthesia and surgical preparation

On the day of experimentation, after the IH protocol, each rat was anesthetized with pentobarbital sodium (70 mg/kg, i.p.) and analgesic agent butorphanol tartrate (0.5 mg/kg, i.p.). Supplementary doses of pentobarbital (∼15 mg/kg/hr i.p.) and butorphanol tartrate (0.025 mg/kg/hr i.p.) were periodically administered to maintain a surgical level of anesthesia. Throughout the experimental protocol, body temperature was maintained at 37°C using a rectal thermistor coupled with a thermostatically controlled heating pad. The trachea was cannulated and the lungs ventilated with a rodent ventilator (SN-480-7, Shinano, Tokyo, Japan). The inspired gas was room air. A femoral vein was cannulated for drug administration. A 20-gauge BD Angiocath catheter (Becton Dickinson Inc., Sandy, Utah), with the tip at a 30-degree angle, was inserted into the jugular vein and advanced into the right ventricle for administering contrast agent.

### SR microangiography

The pulmonary circulation of the anesthetized rat was visualized using SR microangiography at the BL28B2 beam line of the SPring-8 (largest third-generation synchrotron radiation facility, Hyogo, Japan). We have previously described in detail the accuracy and validity of SR for visualizing the pulmonary microcirculation in closed-chest rat [29]. The rat was securely fastened to a clear Perspex surgical plate, which was then fixed in a vertical position in front of the beam pathway, so that the SR beam would pass perpendicular to the sagittal plane from anterior to posterior through the rat thorax and ultimately to a SATICON X-ray camera. The visualised area was 9.5 × 9.5 mm square at the upper part of the left lung.

For each 2-s period of imaging, 100 frames were recorded. During vessel imaging, the three-way stopcock on the right ventricle catheter was opened to a clinical auto-injector (Nemoto Kyorindo, Tokyo, Japan), which was used to inject a single bolus of contrast agent (Iomeron 350; Eisai Co.Ltd., Tokyo, Japan) at high speed (0.4 ml at 0.4 ml/s). Rats were given at least 10 min to recover from each bolus injection of contrast agent.

Following baseline imaging, the rat was exposed to acute hypoxia (10% O_2_) for 5 min and imaging was recorded. After it was re-exposed to room air, upon recovery, atenolol (hydrophilic selective β_1_-blocker, 2 mg/kg, Sigma-Aldrich, St.Louis, MO) was intravenously administered to the rat and imaging was recorded 5 min after injection. Subsequently, to assess the effect of additional blockade of β_2_AR on HPV, imaging in response to the 5 min hypoxic exposure was recorded 5 min after intravenous injection of nadolol (hydrophilic β_1_ and β_2_-blocker, 2 mg/kg, Sigma-Aldrich). The dose of all β-blockers used in this study was based on well-documented recommendations in the literature.

### Chronic administration of β-blockers

N- and IH-rats received atenolol (1mg/kg/day, Sigma-Aldrich) and nadolol (1mg/kg/day, Sigma-Aldrich) infusion from subcutaneously implanted osmotic minipumps for 42 days (model 2006, Alzet, Palo Alto, CA).

### Image Analysis

The computer-imaging program Image Pro-plus ver. 4.1 (Media Cybernetics, Maryland, USA) was used to enhance contrast and the clarity of angiogram images (see Schwenke et al. [28] for a full description). The line-profile function of Image Pro-Plus was used as an accurate method for measuring the internal diameter (ID) of individual vessels [30], [31]. A 50 µm-thick tungsten filament, which had been placed directly across the corner of the detector’s window, appeared in all recorded images and was subsequently used as a reference for calculating vessel diameter (µm), assuming negligible magnification.

### Hemodynamic and ventricular weight measurements

After 6 weeks of IH exposure, rats were anesthetized with pentobarbital i.p. (70 mg/kg). Under mechanical ventilation, right ventricular systolic pressure (RVSP) was measured through a trans-thoracic route using Advantage PV catheter (Scisence, London, Canada) and the data were collected and analyzed using the PowerLab Software (ADInstruments, Castle Hill, Australia). Right ventricular hypertrophy was assessed by the weight ratio of right ventricle/body weight and Fulton’s index, i.e. the weight ratio of right ventricle/left ventricle and septum (R/L+S).

### Immunohistochemistry of Lung Sections

Paraffin blocked lungs from N and IH-rats were sliced into 5-µm sections and placed onto clean glass slides. After deparaffinization, the slides soaked in the citrate buffer were heated with microwave for 5 min for antigen retrieval. Then, the slides were incubated in methanol with 3% hydrogen peroxide for 10 min to block endogenous peroxidase activity. Nonspecific protein binding was blocked by treatment with normal bovine serum albumin for 30 min. The sections were incubated overnight with primary antibodies, anti-β_1_AR antibody (Santa Cruz Biotechnology, California, CA), anti-β_2_AR antibody (Santa Cruz Biotechnology, California) and anti-α-SMA antibody (Sigma-Ardrich, St.Louis, Missouri), at 4°C. The slides were then washed 3 times with PBS and treated with secondary antibodies for 30 min at room temperature. After washing 3 times, the slides were exposed to an ABC horseradish peroxidase (HRP) reagent (Vector Laboratories, Burlingame, CA) in PBS for 30 min. The GFP signal was developed with Peroxidase Substrate Kit AEC (Vector Laboratories) for β_1_AR staining and DAB for β_2_AR staining (Vector Laboratories, Burlingame, CA), and finally the slides were mounted with mounting medium. The stained sections were visualized with an Eclipse E400 microscope (Nikon, Tokyo, Japan) attached to a high-resolution digital camera DXM 1200F (Nikon). Images were captured with ACT-1 software (Nikon).

### Quantitative Analysis of Pulmonary Arteries

Quantitative image analysis of immunohistochemical stained sections with anti-β_1_AR and anti-β_2_AR antibody was performed with Image Pro-Plus ver.4.1 software as described previously [32], [33]. The positively stained area was selected semi-automatically and optical density and area were obtained automatically. Quantification of the expression level of the protein was estimated as expression level score (ELS) : ELS = (mean optical density of positively stained area – mean optical density of background area) x percent area of positively stained area.

### Morphometric Analysis of Pulmonary Arteries

Pulmonary vascular remodeling was assessed by measuring medial wall thickness in vessels of diameter 50 to 150 µm with immunohistochemical staining of anti-α-SMA antibody as described previously [34]. At least 10 representative pulmonary arterioles were chosen from the left lobe of each animal. Morphometric analysis of medial wall thickness was performed using the software Image Pro-Plus ver. 4.1 (Media Cybernetics). Percent medial wall thickness was calculated as the medial wall thickness (the distance between the internal and external lamina) divided by the diameter of the vessel (the distance between the external lamina) x 100.

### Western blot analysis

For Western blotting, frozen lung tissue (0.1 g) was homogenized with 1 mL of ice-cold RIPA buffer (0.1% SDS, 0.5% DOC, 1% NP-40, 150 mM NaCl, 50 mM Tris-Cl pH 7.4) containing 50 mM NaF, 1 mM Na_3_VO_4_ and Complete Protease Inhibitor Cocktail (Roche Diagnostics, Mannheim, Germany). To remove debris, the homogenate was centrifuged at 5.500×g for 10 min, and supernatant was used for analysis, and the rest was frozen at −80°C. The homogenate was subjected to SDS-PAGE on a 4–20% gradient precast gel (Bio-Rad, Tokyo, Japan), and separated protein was then transferred to polyvinylidene fluoride (PVDF) membranes using a transfer system Trans Blot Turbo (BioRad, Tokyo, Japan). Nonspecific antibody binding was blocked using 3% skim milk in TBS-T 0.1%, and the membranes were incubated with primary antibodies. The signals were detected by a luminescent image analyzer Image Quant Las 4000 mini (GE Healthcare Japan, Chiba, Japan) using appropriate secondary antibodies coupled to horseradish peroxidase (Promega, Madison, WI). Primary antibodies were anti-β_1_AR antibody (Santa Cruz Biotechnology, Calfornia, CA), anti-β_2_AR antibodies (Santa Cruz Biotechnology), anti-phospho-PI3 kinase 85p (Tyr458)/p55 (Tyr199) antibody (Cell Signaling Technology, Beverly, MA), anti-PI3 kinase p85 antibody (Cell Signaling Technology), anti-phospho-Akt antibody (Cell Signaling Technology), anti-Akt antibody (Cell Signaling Technology), anti-eNOS (ps1177) antibody (BD Biosciences, San Jose, CA), and anti-eNOS/NOS Type III antibody (BD Biosciences).

### Statistical analysis

All statistical analyses were conducted using GraphPad Prism6 (GraphPad Software, Inc., San Diego, CA). The results of angiography are presented as mean ± standard error of the mean (S.E.M.). All other results are presented as mean ± standard deviation (S.D.). The data analysis of hemodynamic and ventricular weight in rats following chronic administration of atenolol and nadolol were performed using two-way ANOVA with Sidak’s multiple comparison tests. The data analysis of Western blot in the experiments with chronic administration of atenolol and nadolol was performed by one-way ANOVA with Dunnett’s multiple comparison tests to compare the expression level of proteins between the IH-group and each of the other groups. All other data analysis was performed using Student’s *t*-test (unpaired). A *P* value of <0.05 was predetermined as the level of significance for all statistical analysis.

## Results

### Neither right ventricular hypertrophy nor RVSP elevation was observed after 6 weeks of IH exposure

**Figure 1:**
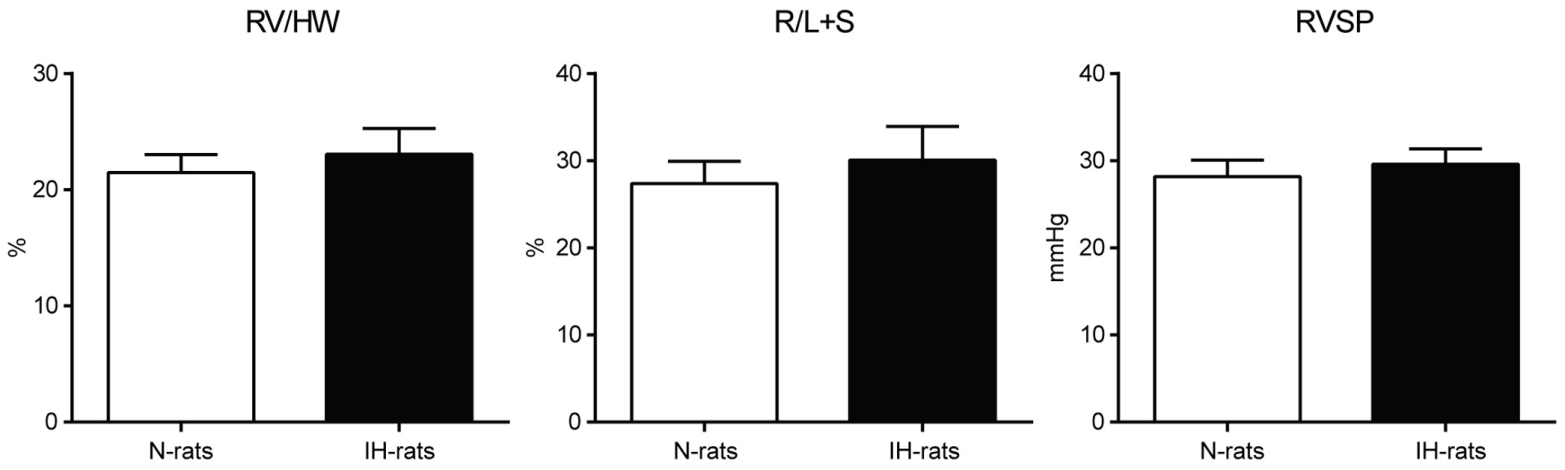
Neither right ventricular hypertrophy nor RVSP elevation was observed after 6 weeks of IH exposure. Heart weight and right ventricular systolic pressure (RVSP) were measured after 6 weeks IH exposure (n = 10). Data are presented as mean ± S.D. RV: right ventricle weight, HW: heart weight, R/L+S (Fulton’s index): right ventricle weight/left ventricle and septum weight.

The right ventricular (RV) weight/heart weight (HW) and Fulton’s index (R/L+S) were not different between N and IH-rats after 6 weeks of experiments. The right ventricular systolic pressure (RVSP) also showed no difference between N and IH-rats under the anesthetic condition. These results mean that IH at a rate of 3 min cycles of 4–21% O_2_ for 8 h/d for 6w does not induce PAH and RVH (Fig. 1).

### HPV is attenuated in IH-rats

**Figure 2:**
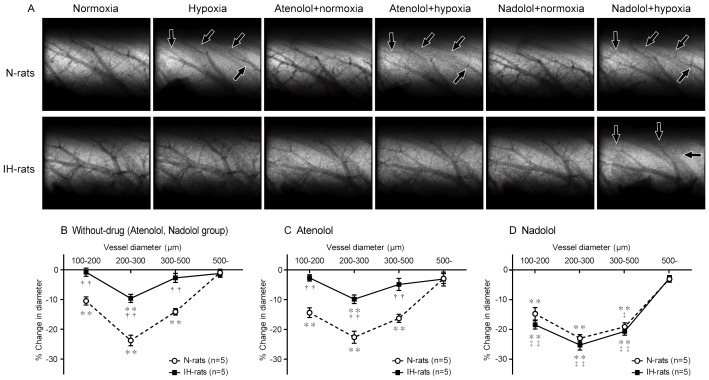
Selective blockade of peripheral β_2_AR restored HPV in IH-rats. (A) Representative microangiogram images showing the branching pattern of small pulmonary arteries during normoxia and in response to hypoxia with or without drugs. Black arrows point to branches of pulmonary arteries that have constricted in response to acute hypoxia. IH has no response to hypoxia, however, significant vasoconstriction is revealed with nadolol. The tungsten wire in the bottom right of each image is a reference of 50 µm diameter. (B) Relationship between vessel size and the magnitude of pulmonary vasoconstriction (% decrease in vessel diameter) in response to acute hypoxia (10% O_2_ for 10 min) in N-rats and IH-rats with or without β-blocker administration. Data are presented as mean ± S.E.M. ^*^Significant reduction in vessel diameter compared to normoxic condition (^**^ *P*<0.01). ^†^Significant difference between N-rats and IH-rats (^††^ *P*<0.01).^ ‡^Significant difference compared to without-drug. (^‡‡^ *P*<0.01).

In N-rats, acute hypoxic exposure (10% O_2_) induced clear ID constriction (HPV) in the small pulmonary arteries with ID of 100–500 µm, but not in those more than 500 µm (Fig. 2A, B). The magnitude of HPV (% reduction of ID with acute hypoxia) tended to increase as arterial diameter decreased, with the greatest degree of constriction (approximately 24%) occurring in those arteries with ID between 200 and 300 µm. In IH-rats, significant vasoconstriction was only evident in the 200–300 µm pulmonary vessels. Moreover, the degree of vasoconstriction was only approximately half of that in N-rats. All other arteries/arterioles with an ID less than 500 µm did not significantly constrict, meaning that the magnitude of HPV was greatly attenuated in IH-rats (Fig. 2A, B).

### Selective blockade of peripheral β_2_AR restored HPV in IH-rats

Pretreatment with atenolol (hydrophilic peripherally acting β_1_-blocker) did not affect the baseline internal diameter and HPV level in all 100–500 µm pulmonary arteries in N- and IH-rats (Fig. S1, Fig. 2A, C). After atenolol administration, nadolol (hydrophilic peripherally acting β_1_ and β_2_-blocker) was administered to inhibit β_1_ and β_2_AR completely. Nadolol administration also had no significant effect in the baseline internal diameter of pulmonary arteries in N- and IH-rats. (Fig. S1). However, nadolol significantly augmented HPV in IH-rats so that the magnitude of HPV of all 100–500 µm arteries was similar to that observed in N-rats, although it had no significant effect on HPV in N-rats (Fig. 2A, D). These results suggest that HPV was attenuated by the activation of peripheral β_2_AR during IH.

### Chronic administration of nadolol induced PAH and RVH in IH-rats without pulmonary arterial hypertrophy

**Figure 3:**
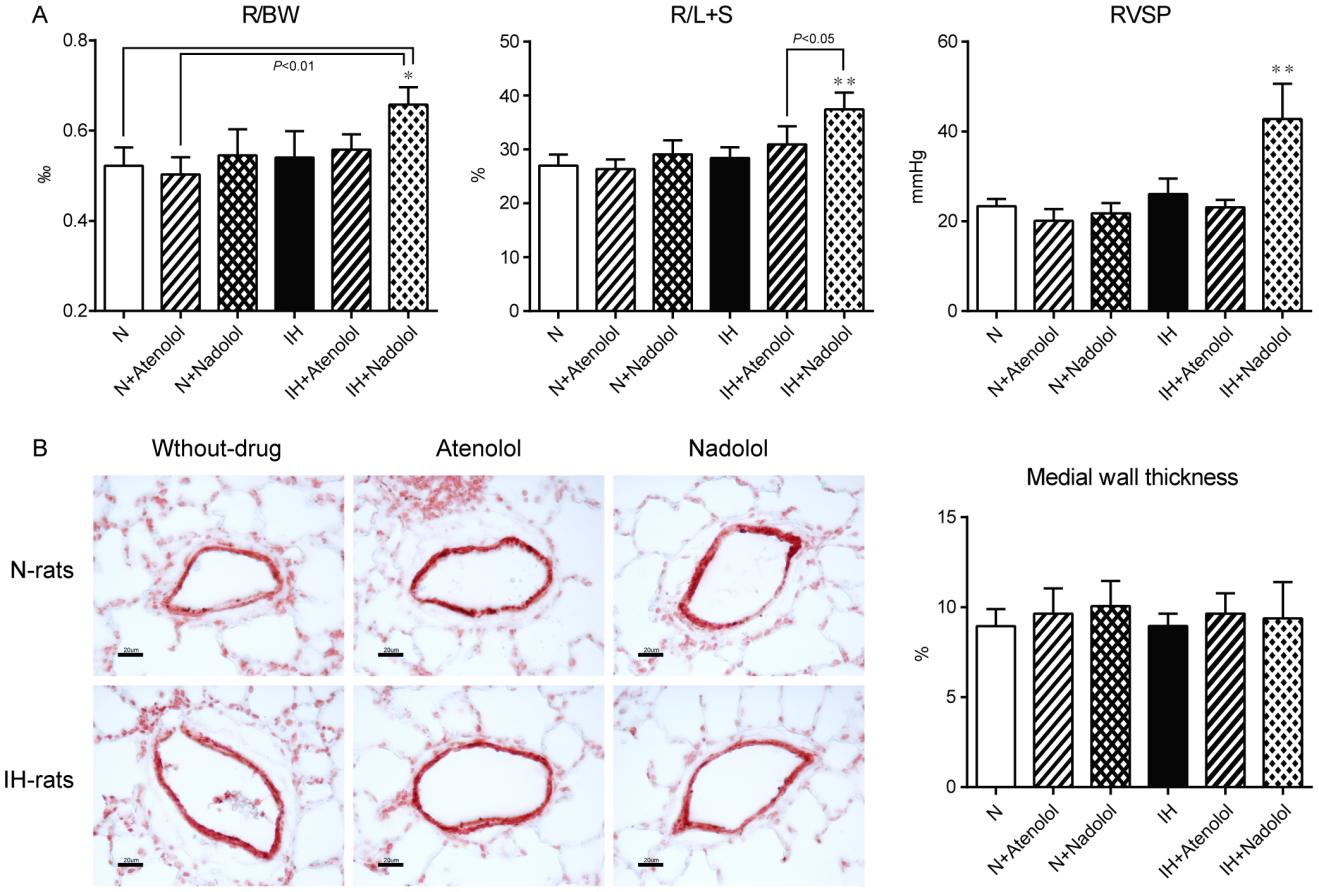
Chronic administration of nadolol induced PAH and RVH in IH-rats without pulmonary arterial hypertrophy. (A) Hemodynamic and morphometric change after chronic subcutaneous administration of atenolol and nadolol (n = 4 each) during 6weeks of IH exposure. Data are presented as mean ± S.D. ^*^Significant change compared with every other group (^*^ *P*<0.05, ^**^ *P*<0.01). (B) Representative images of small pulmonary arteries and assessment of pulmonary arterial hypertrophy by means of medial wall thickness in N and IH-rats with/without chronic administration of atenolol or nadolol (n = 4 each). There were no significant differences in medial wall thickness between each group. Calibration bar = 20 µm. Data are presented as mean ± S.D.

To confirm that activation of β_2_AR dependent attenuation of HPV leads to prevention of the development of PAH and RVH, nadolol was administered chronically using osmotic minipumps for 6 weeks of IH exposure. The results show that the weight of right ventricle and RVSP were significantly increased in IH-rats with nadolol administration, meanwhile, nadolol had no effect in N-rats (Fig. 3A). On the other hand, chronic administration of atenolol had no effect in both N and IH-rats. The immunohistochemistry of the lungs of these rats show that there was no pulmonary arterial hypertrophy (increase in medial wall thickness) in IH (Fig. 3B).

### Expression level of β_1_AR was decreased and β_2_AR was preserved in the pulmonary arteries of IH-rats

**Figure 4:**
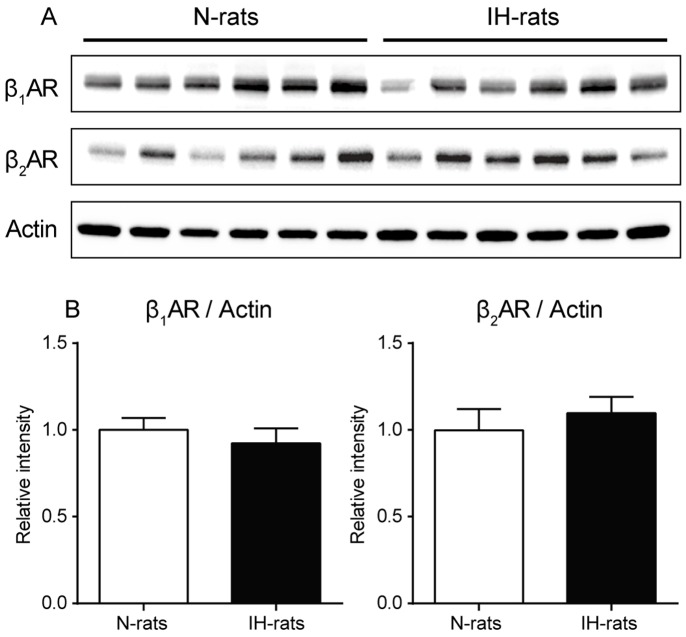
Expression of β_1_ and β_2_AR was slightly changed but not significant so in the lung of IH-rats. Quantitative analysis of β_1_AR, β_2_AR, and actin protein expression in whole lung of N-rats and IH-rats (n = 6 each) using Western blot. (A) representative Western blot bands, (B) relative amount of protein. Data are presented as mean ± S.D.

**Figure 5:**
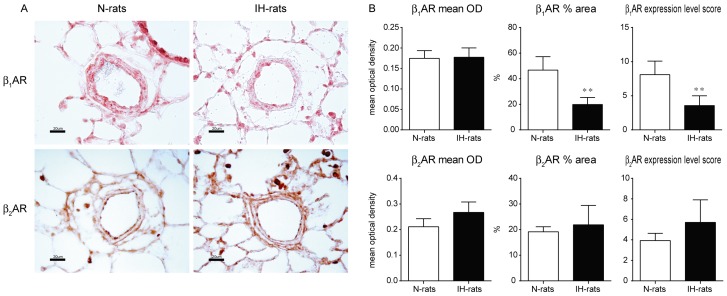
Expression level of β_1_AR was decreased and β_2_AR was preserved in the pulmonary arteries of IH-rats. Quantitative immunohistochemistry of β_1_ and β_2_AR was performed in the pulmonary arteries in the diameter range of 50 to 150 µm (n = 6 each). (A) representative images of immunohistochemistry, (B) mean optical density, % area, and expression level score. Quantification of the expression level of the protein was estimated as expression level score (ELS) : ELS = (mean optical density of positively stained area – mean optical density of background area) x percent area of positively stained. ^*^Significant difference between N and IH-rats (^**^ *P*<0.01).

We evaluated the amount of β_1_ and β_2_AR in the lung using Western blot analysis (Fig. 4). The results show that β_1_AR was slightly decreased and β_2_AR was slightly increased in IH, but there was no significance between N and IH-rats. Therefore, we performed quantitative analysis of immunohistochemistry in pulmonary arteries (Fig. 5). β_1_AR was observed in both endothelium and medial wall of pulmonary arteries. The density of β_1_AR was not different between N and IH-rat, however, % area was apparently decreased in IH-rats, especially in medial wall area. Therefore, the total amount (expression level score) of β_1_AR was significantly decreased in IH. On the other hand, β_2_AR was expressed in endothelium only. The density of β_2_AR tended to increase in IH compared to N-rats, but there was no difference significantly. The area of β_2_AR was not different in both groups. The total amount of β_2_AR tended to increase in IH-rats, but there was no significant difference because the expression level score in IH varied widely.

### IH-induced phosphorylation of p85, Akt and eNOS were abrogated by nadolol administration in the lung tissue of IH-rats

**Figure 6:**
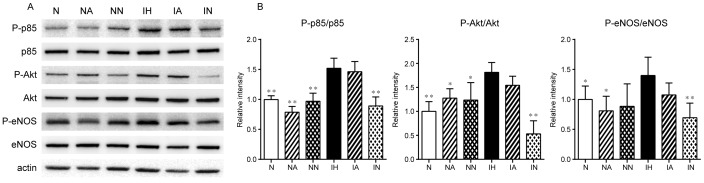
IH-induced phosphorylation of p85, Akt and eNOS were abrogated by nadolol administration in the lung tissue of IH-rats. Quantitative analysis of phospho-p85, p85, phospho-Akt, Akt, phospho-eNOS, eNOS, and actin protein expression in whole lung of N-rats and IH-rats (n = 6 each) using Western blot. (A) representative Western blot bands, (B) relative amount of protein. Data are presented as mean ± S.D. ^*^Significant difference between IH-group and each other groups (^*^ *P*<0.05, ^**^ *P*<0.01). N: N-rats, NA:N-rats+atenolol, NN: N-rats+nadolol, IH: IH-rats, IA: IH-rats+atenolol, IN: IH-rats+nadolol.

We assessed phosphorylated level of p85 (chief component of PI3K), Akt, and eNOS in the lung tissue using Western blot analysis. The results showed that all of these proteins were significantly increased in phosphorylated level in IH compared to N-rats (Fig. 6A, B). Chronic administration of nadolol significantly abrogated the phosphorylation of these proteins, but chronic administration of atenolol did not. Both atenolol and nadolol had no effect in phosphorylation of proteins in N-rats. Actin level was not significantly different between any of the groups.

## Discussion

This study demonstrates that the activation of β_2_AR in the pulmonary arteries attenuate HPV and prevent the development of IH-induced PAH. In IH-rats, the expression level of β_2_AR was preserved at the same level as N-rats, meanwhile, β_1_AR was down-regulated in small pulmonary arteries. The component of PI3 Kinase P85, Akt, and eNOS were all phosphorylated in IH-rats, and these phosphorylation levels were lower as a result of chronic inhibition of β_2_AR, meaning that IH-derived activation of β_2_AR/P85/Akt/eNOS pathway prevents PAH progression. These results suggest a possible explanation for the reason why PAH is not an universal finding in SAS patients and experiments with IH-animals.

It is well documented that in normoxic pulmonary arteries, both the β_1_ and β_2_AR have a vasodilatory effect [17]–[19]. In the isolated perfused lung, both β_1_ and β_2_AR have a potency to attenuate HPV in normal rats [35], [36]. In the present study, the combined blockade of β_1_ and β_2_AR (nadolol) restored acute HPV in IH-rats, but not in N-rats. Meanwhile, the selective blockade of β_1_AR (atenolol) did not modify HPV in either IH or N-rats. Considering that atenolol and nadolol are hydrophilic and difficult to pass through the blood-brain barrier, these data suggest that the peripheral pulmonary β_2_AR mediated vasodilator mechanism was activated by IH and played an important role in attenuating of acute HPV. Meanwhile, the β_1_AR dependent vasodilator mechanism did not contribute to attenuate HPV.

In this study, perfusion pressure (i.e. pulmonary arterial pressure) and flow (i.e. cardiac output) are not reported. It is likely that βAR blockade (with atenolol or nadolol) reduced cardiac output [37], [38]. Therefore, it may be expected that the vasoconstriction capacity of the pulmonary vessels is influenced by the changes in pulmonary pressure and cardiac output. However, our previous study reported the vasoconstrictor capacity of the pulmonary vessels is relatively independent of changes in cardiac output and perfusion pressure [20]. Indeed, we demonstrated that, when the increase in PAP during acute hypoxia is prevented by experimentally reducing cardiac output, there is a tendency for the decrease in vascular diameter to shift downward (i.e. the vasoconstriction is equally altered for all vessel sizes); however, since no statistical difference was evident in the magnitude of vasoconstriction in this study when comparing constant pulmonary arterial pressure with the usual pressure rise, the effect of pulmonary pressure and cardiac output on the magnitude of acute hypoxic vasoconstriction is likely to be negligibly small.

The chronic administration of nadolol induced PAH and RVH in IH-rats, but atenolol did not. Notably, pulmonary arterial remodeling was not observed in IH-derived PAH. These results indicate that the pulmonary arterial hypercontraction derived from repeated HPV is a chief cause of the IH-induced PAH, and the β_2_AR-mediated vasodilator mechanism of the pulmonary arteries continuously attenuates HPV during IH exposure.

We suggest that HPV probably chiefly contributes to increases in pulmonary arterial pressure during IH, however, it is possible that a HPV-independent vasoconstrictor mechanism also contributes to increases in pulmonary arterial pressure. Data from both patients and animals suggest that IH can upregulate vasoconstrictors such as endothelin-1 (ET-1). Plasma concentrations of ET-1 were increased in patients with SAS [39] and rats exposed to IH [40]. CPAP therapy in patients with SAS show reduced plasma ET-1 levels [39]. In a human study, chronic administration of atenolol for 22 weeks significantly decreased mean blood pressure and blood ET-1 concentration in patients with systemic hypertension [41]. Meanwhile, urine concentration of prostacyclin metabolites was significantly higher in untreated SAS patients than in controls, and 3 days of CPAP therapy normalized its concentration with a significant decrease in mean blood pressure, probably meaning that the production of prostacyclin plays a role in compensating for the systemic hypertension in SAS [42]. Chronic administration of atenolol for 2 weeks promoted prostacyclin secretion from the aortic wall in spontaneously hypertensive rats [43]. Nadolol increased prostacyclin-induced bronchodilation via increased expression of prostacyclin receptors in bronchial smooth muscle cells in a murine asthma model [44]. These results suggest that the possibly other vasoconstrictors and even vasodilators may have relationship in progression of cardiovascular disorder in SAS. As atenolol and nadolol have significant effects on these factors, additional research is needed to reveal the contribution of these possible factors to PAH in IH.

In the SR pulmonary angiography protocol, we used intravenous atenolol and nadolol at a dose of 2 mg/kg. In a previous study, atenolol 2 mg/kg i.v. blocked cardiac chronotropic and inotropic responses evoked by unilateral left- or right-dorsomedial hypothalamus activation in rats [45]. Nadolol 2 mg/kg i.v. had significant inhibitory effect on phentolamine-induced dilation of coronary artery in dogs [46]. These results indicate that 2 mg/kg i.v. of atenenolol and nadolol have physiologically significant effects on cardiovascular function. In the present study, neither atenolol nor nadolol changed the baseline of internal diameter in the pulmonary arteries in N and IH-rats. This suggests that atenolol and nadolol *per se* at the dose of 2 mg/kg i.v. have no direct vasomotor effect on the pulmonary arteries under the normoxic condition.

For chronic administration of atenolol and nadolol, we used a dose of 1 mg/kg/day with osmotic minipumps for 6 weeks. Atenolol 1 mg/kg/day for 28 days prevented the increase in both βAR-kinase expression and activity in the heart induced by chronic activation of sympathetic nervous system due to feeding of a low-sodium (0.05%) diet in rats [47]. Nadolol 1 mg/kg/day for 7 days enhanced both splenic and peritoneal T_CD8+_ cell activation which was limited by activation of the sympathetic nervous system in mice [48]. In our experiments, there was a limit on the amount of drugs that could be administered, since we had to employ a small type of minipump for subcutaneous implantation into 6-week-old rats. Therefore, we determined a minimum and effective dose of atenolol and nadolol in reference to these previous reports for chronic experiments.

In immunohistochemistry, β_1_AR was expressed in both endothelium and medial wall while β_2_AR was observed only in endothelium in pulmonary arteries. These results correspond to a previous report [17]. Quantitative immunohistochemistry revealed that the expression of β_1_AR was apparently decreased, and β_2_AR was preserved in pulmonary arteries in IH. Notably, the density of β_1_AR was not different between N and IH-rats, but the expression area of β_1_AR was decreased especially in the medial wall in IH. On the other hand, the Western blot analysis showed the expression level of protein in β_1_ and β_2_AR was not significantly changed in the lung tissue of IH-rats. The discrepancy in β_1_AR expression between the Western blot data and the immunohistochemistry data suggests pulmonary arterial β_1_AR is locally down-regulated in IH-rats.

In the present study, Western blots showed that p85, Akt, and eNOS were all significantly phosphorylated in the lung tissue of IH-rats. These phosphorylation levels were abrogated by chronic administration of nadolol in IH, but not by atenolol. Prolonged activation of β_2_AR switches G-protein from Gs to Gi [27]. β_2_AR-Gi signaling activates PI3K/Akt pathway and promotes eNOS phosphorylation to increase NO release, presumably inducing direct vasodilation in pulmonary artery [17]. Thus, considering the present results, it is suggested that the activation of β_2_AR-Gi-dependent PI3K/Akt/eNOS signaling pathway contributes to attenuate the HPV, leading to the prevention of the development of PAH in IH-rats.

Meanwhile, expression level and sensitivity of βAR subtypes should be strongly influenced by the degree and the duration of hypercatecholemia depending on the duration and severity of hypoxia in IH. Therefore, the down-regulation of β_1_AR protein and the concurrent disappearance of β_1_AR-mediated pulmonary dilation in the present study may depend on the IH conditions e.g. repeatedly alternating between exposure of FiO_2_ 4% and 21% for 90 sec each for 8 h/day during six consecutive weeks. Moreover, we propose that any discrepancy between previous studies which have reported mild [8]–[10], [12] or no [11], [12] RVH associated with IH-rats may be explained by different activation levels of the HPV-attenuation of the βAR mechanism depending on the degree and duration of hypoxia, the daily duration of IH exposure, and experimental period. This being the case, we have previously reported that rats exposed to chronic sustained hypoxia develop severe PAH, yet still appear to a normal HPV [49]. Further, assuming that IH is a major pathophysiologic basis for SAS [6], the low prevalence of PAH (∼20%) in SAS [1], [4] may be explained by the inhibition of PAH and RVH progress due to the HPV-attenuating βAR mechanism.

In summary, SR microangiography provides direct evidence that the HPV is strongly suppressed by activation of pulmonary β_2_-mediated vasodilation in IH-rats. Neither PAH nor RVH were evident following IH exposure for 6weeks. However, chronic blockade of pulmonary β_2_AR revealed IH-induced PAH and RVH. The activation of β_2_AR-Gi-dependent PI3K/Akt/eNOS signaling pathway presumably contributes to attenuate the HPV, leading to the prevention of the development of PAH in IH-rats. These findings indicate that IH-derived activation of β_2_AR in the pulmonary arteries plays an important role in protecting the pulmonary circulation under the IH condition.

## Supporting Information

Figure S1 The effect of administration of atenolol and nadolol to the internal diameter of small pulmonary arteries (n = 5 each). Data are presented as mean ± S.D.
(TIF)
