# Supplementary figures and images for: β2-Adrenergic Receptor-Dependent Attenuation of Hypoxic Pulmonary Vasoconstriction Prevents Progression of Pulmonary Arterial Hypertension in Intermittent Hypoxic Rats
Source: PLoS One. 2014 Oct 28;9(10):e110693. doi: 10.1371/journal.pone.0110693 (PMC4211686; doi:10.1371/journal.pone.0110693)

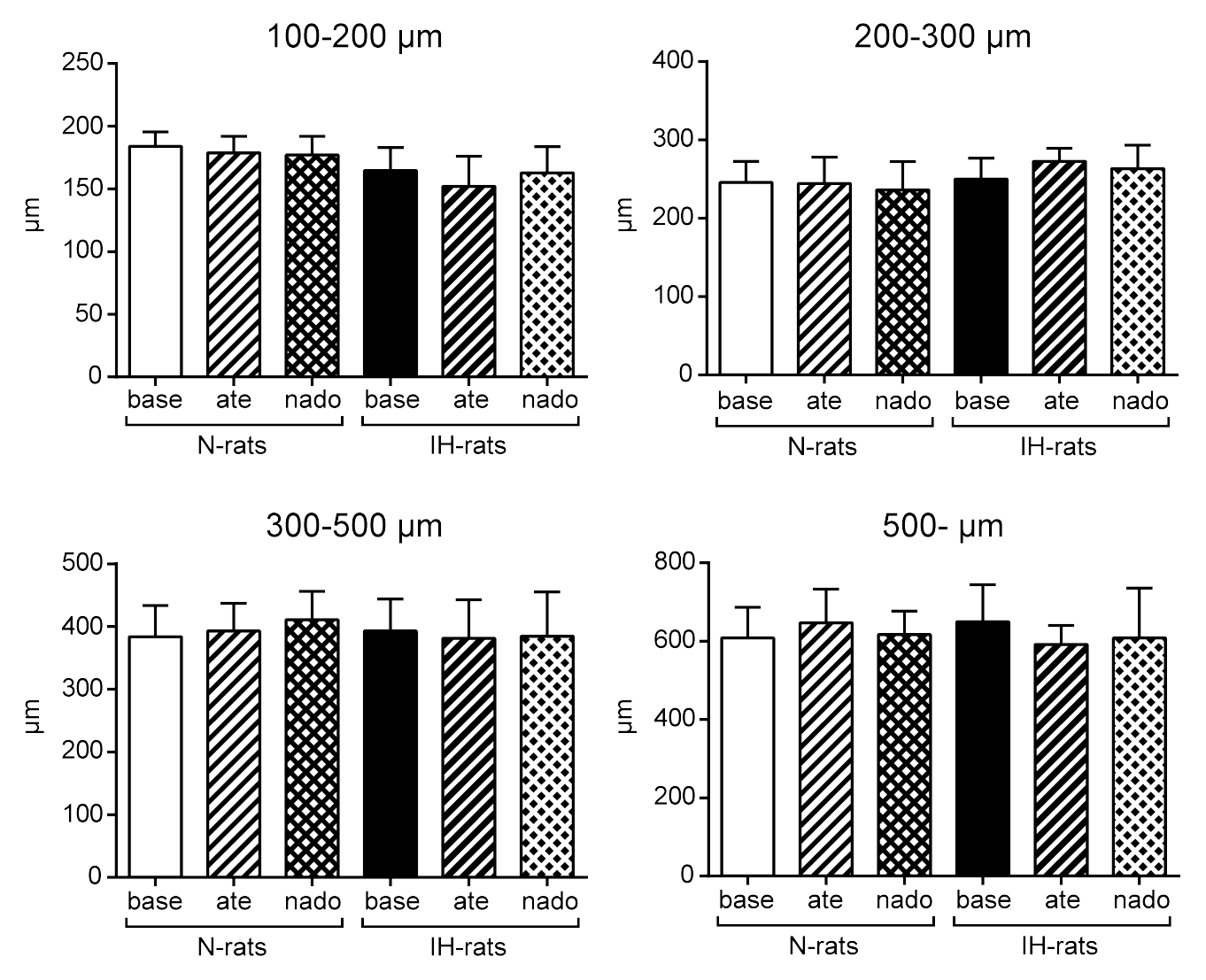

Supplement: Figure S1 — The effect of administration of atenolol and nadolol to the internal diameter of small pulmonary arteries (n = 5 each). Data are presented as mean ± S.D. (TIF) [file pone.0110693.s001.tif]
